# Aretæus of the Causes and Signs of Acute and Chronic Disease

**Published:** 1837-10

**Authors:** 


Art. II.—*Aretæus of the Causes and Signs of Acute and Chronic Disease.* Translated from the Greek, by T. F. Reynolds, m.b. f.l s. &c.—*London*, 1837. 8vo. pp. 157.

We have here a translation of those parts of Aretæus which are used for examination at Cambridge and the College of Physicians. Being thus intended for the assistance of students, it has been made extremely literal, perhaps too much so occasionally; but, when taken in this limited point of view, the fault is certainly on the right side. As it *was* written with this intent, we wish that Dr. Reynolds had given the Greek text also, (copies of Aretæus not being very cheap or common;) and added some notes, as his author is by no means one "whom he that runs may read."

As examples of Aretæus's manner of treating his subjects, and as specimens of the translation, we extract the following passages, relating to two of our best known diseases, one acute and one chronic. Our readers will not fail to observe, in these extracts, defects of expression, of punctuation, and even of grammar.

"*On Cholera*. Cholera is a retrograde motion of the material of the whole frame into the œsophagus, stomach, and intestines, and is a very severe disorder. What is accumulated in the œsophagus runs off by vomiting, while what is in the stomach and intestines passes below. The first discharges that are thrown off by sickness have a watery look, while those by stool are fluid, stercoraceous, and offensive. This disease is caused by continued crudities, which, when they are washed out, are followed first by slimy, then by bilious evacuations: the first are readily brought away without pain. Afterwards there is tightness in the œsophagus and griping in the belly. If the disorder get worse, the gripings become severer, fainting supervenes, the limbs refuse their support, there is anxiety, and loathing of food; or if any be taken, it is attended with the vomiting of bile of an exceedingly yellow colour, which comes away with a gush and nausea; the stools have the same appearance, while spasms and cramps arise in the calves of the legs and in the arms; the fingers become bent; dizziness and hiccough come on; the nails are livid, with a sense of great cold at the extremities and general shivering. If the disorder be near its close, the patient becomes bathed in sweat; black bile is discharged upwards and by stool, while, from the bladder being spasmodically contracted, the urine does not pass away, and is not even secreted, from the fluids being diverted to the intestines. Speech is lost, pulse very small and frequent, as in syncope; the efforts to vomit are incessant and ineffectual; there is constant desire to go to stool, with tenesmus, but the effort is unavailing, and no liquid flows. Death is very painful and rapid, accompanied with spasm, choking, and retching. Summer is the time for this disease, and then the autumn; the spring brings it less frequently, the winter least of all. The periods of life liable to it are youth and manhood; old age is less so than any other time of life. Children are more liable to it, but it does not prove deadly." (P. 37.)

"*On Phthisis*. . . . The varieties of sputa are infinite, livid, black, or very dark, pale, white, or whitish green, flat or round, hard and tenacious, or thin, and soluble; free from smell or offensive; all these varieties does the pus assume. They who try the sputa, either by fire or water, do not seem to me to understand Phthoë, for the sight is more to be depended on than all the other senses together, not merely in respect to what is brought up, but also with reference to the appearance of the patient, for when any person sees another pale, feeble, constantly coughing and wasting away, he pronounces it a clear case of phthoë." . . . "The voice is hoarse, the neck slightly twisted, elongated, and not easily turned to either side, being as it were somewhat stretched, the fingers are lean, though their joints appear thick, they seem mere bones, so much has the flesh shrunk away, the nails are adunced; the abdomen is wrinkled and flat, for from loss of flesh it neither preserves its filleting nor rotundity, for the same reason the nails become curved, for the fulness of the finger ends, that used to expand and support them, have become, as it were, solid, which causes them to feel painful; the nose is pointed, the cheeks flushed and prominent, the eyes are sunken, but sparkle with a liquid lustre, the face is puffy, pale or livid, the thin parts about the jaws stretched on the teeth, the patients wear a ghastly smile, and their whole appearance is of this character, they are emaciated and fleshless, the muscles of their arms are indistinct, with no vestige of breasts, the nipple merely is visible, you may not merely readily count the ribs, but see where they end, for not even are their articulations with the vertebræ well concealed nor where they overlap the sternum; the interval between them is hollow and curved; the hypochondria are sunk and retracted, the epigastrium and flank adhere as it were to the spine. The joints of the leg, hip, and arm become conspicuous, prominent, and devoid of flesh, and the spines of the vertebræ which were originally in a hollow, now project from the wasting of the muscles on each side, the shoulderblades are seen jutting out like birds' wings. If in such a case the bowels become disordered, it is hopeless." (P. 78.)

Dr. John Clarke in his commentaries on the Diseases of Children, gives Aretæus the credit of having known that, in the first stage of phrenitis, the pupil is contracted. He says "In the early period of the author's practice, he thought that this symptom had not been before observed, as it was not described by modern authors upon the complaint; but, upon consulting Aretæus, *περι παραλυσεως*, it appears that he had observed the symptom, and he also describes it as belonging to the early stage of the disease, and has made a marked distinction between the state of the pupil of the eye, in the commencement and advanced states of it." (*Commentaries*, p. 121-2.)

Aretæus does not, however, make the distinction that Dr. Clarke supposes; nor is he speaking of inflammation of the brain, but merely of paralysis. The passage in question is thus translated by Dr. Reynolds:

"The pupil of the eye is liable to both these varieties of disease, for it becomes greatly dilated, and the affection is termed platucoria, and sometimes contracted to a very small size, which I term phthisis, or mudriasis; the bladder also suffers a paralysis of the functions peculiar to it, being either paralyzed, in a state of distention, or becoming incontinent, or rolled up as it were in itself when full of urine, and unable to void it." (P. 75.)

Dr. Clarke quotes the original passage in a sadly distorted state : it is to be found at p. 40 of Wigan's edition.

One of the most curious things in Aretæus is, that he not only knew that the arteries contained blood, but recommended bleeding from those behind the ear, as a cure for headach. He says, they may be distinguished by their pulsation. (*De Morb. Diut. cur.* lib. i. cap. 2.)

